# Foreign

**Published:** 1841-10-16

**Authors:** 


### FOREIGN.

*Clinical Lecture, delivered at University College Hospital.* By Samuel Cooper, Esq., Senior Surgeon to the Hospital, &c.

*Gentlemen,*—I invite your attention to two examples of injury of the elbow at present in the hospital; one, attended with circumstances perhaps absolutely preventive of a complete cure; the other, generally sure to have a favourable termination under proper treatment.

Case I.—*Dislocation of the ulna from the humerus upwards and outwards, with fracture or separation of the coronoid process. The case of three months' standing. Division of the tendon of the triceps.*

Lucy Clayton, æt. 14, admitted March 16, 1841. Last Christmas-day she slipped and fell down on the ice, and hurt her elbow, though her hand first came against the ice, in the attempt, which she had made, to save herself from harm. Directly after the accident she was unable to bend the arm, which was fixed almost in the extended position. At the same time, a good deal of pain was complained of in the shoulder, and the forearm was benumbed. The elbow, which was much deformed, soon swelled considerably. Five hours after the accident, a surgeon, who saw the case, pronounced it to be a dislocation, and, after making extension and counter-extension, he applied a lotion of vinegar and water.

On her admission into this hospital, about twelve weeks after the occurrence, the joint presented a very deformed appearance, and its mobility was but slight. The inner condyle made a remarkable prominence, between which and the displaced ulna, an unusual depression was perceptible. The radius rotated freely. The point of the olecranon, which projected upwards and backwards, was about half an inch further from the inner condyle, than the corresponding point of bone in the other arm was from the same part of the humerus in that limb. Whenever an attempt was made to bend the arm, the triceps was rendered exceedingly tense. In front, a firm substance, which could be moved about, was felt; and concluded to be the coronoid process detached from the rest of the ulna.

March 18.—Attempt made to bring the ulna into a better position. After extension and counter-extension had been maintained for some time, I forcibly bent the arm over my knee. Mr. Quain then repeated the same proceedings, with the view of bringing the ulna into its proper position, and immediately afterwards the girl was able to put her hand up to her forehead, which she had not been able to do since the accident. This amendment, however, did not continue in the degree here described. Hence, for several weeks, a mechanical apparatus has been employed for the purpose of maintaining and gradually increasing the flexion of the elbow. Together with the use of this instrument, we have now begun to combine the exercise of passive motion for a certain time almost every day. At present the girl undoubtedly has more use of her arm, than when she was first admitted; yet, her own power of bending the elbow, it must be confessed, is very limited, and, if passive motion of it were not still rigorously followed up for some time, I should be apprehensive of an anchylosis between the ulna and the humerus.

When the ulna is displaced backwards, and the coronoid process is broken off, or detached, in a young subject from the rest of the bone, you know perfectly well that the maintenance of the reduction is much more difficult, than when no such complication exists.

In Sir Astley Cooper's work on Dislocations, I find the following observations under the head of Fracture of the Coronoid Process of the Ulna; and they are interesting in relation to the accident now engaging our attention. A gentleman, after falling upon his hand, found himself unable to bend the elbow; nor could he entirely straighten it. The same things happened in Lucy Clayton.

"He applied to his surgeon, who, upon examination, found that the ulna projected considerably backwards; but that, as soon as he bent the arm, it resumed its natural form. He immediately confined the limb in a splint, and kept it in a sling." When Sir Astley Cooper saw this patient in town, several months had elapsed since the accident; yet the same appearances, which the surgeon described as presenting themselves when he first saw the injury, still remained; namely, the ulna projected backwards whilst the arm was extended; but it could be drawn forwards and bent, without much difficulty, and the deformity was then removed.

Sir Astley Cooper had been for some years accustomed to mention this case in his lectures, when a subject was brought to the dissecting room, who had met with the same accident. The coronoid process, which had been broken off within the joint, had united only by ligament, so as to move readily upon the ulna, and thus the sigmoid cavity was so altered, that in extension the ulna glided backwards on the humerus. Sir Astley Cooper doubted whether any treatment of such an accident would perfectly succeed, because the coronoid process does not admit of bony union. He recommended, however, the arm to be kept steadily in the bent position for three weeks after the injury, so as to render the ligamentous union as short as possible.

As in our case the triceps was remarked to become exceedingly tense, whenever an attempt was made to bend the arm, Mr. Quain decided to try what benefit would result from dividing the tendon of that muscle, which operation was performed about three weeks before the girl left the hospital, during most of which time the instrument was also worn a part of the day for the purpose of keeping the limb bent. This treatment was certainly productive of some good, though the power of bending the elbow is yet very limited. She has been directed to continue the use of the splint for some hours every day, in order gradually to increase the flexion of the limb by means of the screw; and to let the joint be bent and extended by other persons for twenty minutes or half an hour daily, when the instrument is not on the limb. I think that, in this manner, she will ultimately have a good use of the arm.

Case II.—*Fracture of the internal condyle of the humerus.*

Robert Fisher, æt. 12, admitted March 25th. In running across the street, his foot touched the curb-stone, and he fell with his elbow against the pavement.

On his arrival at the hospital the elbow was found to be exceedingly painful and much swollen—flexion and extension could be performed, but not without severe pain. The olecranon did not project backwards more than natural. Pronation and supination could be performed without assistance; and scarcely any deformity was observable except the swelling. On making pressure on the internal condyle, a crepitus was felt. This process was drawn downwards, and was not on the same level with the external condyle. The styloid processes of the radius and ulna bore their normal relative position to each other.

Fomentations were at first applied.

29th.—The swelling and pain are diminished; but considerable ecchymosis is seen.

31st.—The ecchymosis yet continues; pain is felt in the hand, and in the ring and little finger, especially when the arm is extended.

April 1st.—This pain is yet experienced. Two lateral angular splints have been applied to the limb, which is kept bent at right angles.

10th.—Splints taken off and reapplied. Fractured condyle in good position. The swelling and ecchymosis have subsided. Flexion of the arm causes pain.

22d.—Less pain felt on moving the joint. Still some pain in the hand, the ring, and little finger.

May 4th.—Splints readjusted. Patient discharged.

The particulars of this case make us acquainted with one symptom of a fracture of the inner condyle, which, though from anatomical considerations it might be expected generally to attend such an accident, has not, I believe, been noticed by surgical writers. I allude to the pain felt by the patient in the ring and little fingers, and in the palm of the hand, evidently arising from dilatation or disturbance of that part of the ulnar nerve which lies between the olecranon and internal condyle. You know that when a nerve is irritated, or disturbed in its course, the principal pain is usually felt in the ultimate divisions of it. Thus, in a case of popliteal aneurism, the pressure of the tumor on the popliteal nerve usually causes the greatest degree of pain in the parts to which the external and internal plantar nerves are distributed; and in a fracture of the inner condyle of the humerus, if the displacement of the fragment irritated or disturbed the trunk of the ulnar nerve behind it, the pain would be chiefly experienced in the ring and little fingers, and in the palm, where its deeper branch is distributed to some of the smaller muscles of the hand.

From these accidents I will proceed to notice a case of a different nature.

Case III.—*Wound of the Brachial Artery. Apoplexy.*

Matthew West, æt. 49; admitted under Mr. Quain, April 8, 1841.

On returning home this morning, he was seen leaning against a lamp-post, and was unable to answer any questions; but from papers in his pocket, the place of his residence was ascertained, and he was conveyed home in a cab.

A surgeon was sent for, who attempted first to bleed him in the cephalic vein, and then in the median basilic, in doing which the brachial artery was penetrated. A tourniquet was applied, and the patient brought to the hospital. In consultation with Mr. Quain, it was decided, that, as the opening in the artery was large, and the limb paralytic, it would be better in this case not to try pressure, but to tie the wounded part of the artery without delay. While pressure was made on the artery in the middle of the arm by an assistant, Mr. Quain, therefore, enlarged the wound, upwards and downwards, and outwards. The fascia sent off from the tendon of the biceps having then been divided, the wound in the artery was exposed, and a ligature applied above and below it.

The wound was then covered with the water dressing, and the patient put to bed.

He had lost about lbjss. or lbij. of blood from the accident.

April 13th.—Died this evening, five days after the apoplectic attack and the operation. The particulars of the case from the period of the operation until that of the fatal termination are interesting, but, as not bearing upon surgery, I now omit them. Still I recommend the perusal of them to you, as they are recorded in the hospital book.

Some of the post-mortem appearances, however, I will notice.

*Head.*—On opening the dura mater about ℥ij. of serous fluid escaped. The arachnoid membrane was much thickened, opaque, and studded with numerous little tubercles of lymph, over the superior part of the hemispheres.

Under the arachnoid membrane there was also a copious effusion of serum. The arachnoid and the pia mater between the hemispheres were more vascular than natural.

In the right ventricle bloody serum was observed. In the left, a large coagulum was found, occupying all the surface of the thalamus opticus and corpus striatum, which parts were dissolved into a soft bloody pulpy mass.

The arteries at the base of the brain were thickened, but not ossified.

The chief points of instruction afforded by this case relate—

1st, To the necessity of using great caution whenever you attempt to bleed in the median basilic vein, because, if the patient be in a fit at the time, drunk, or from any cause disposed to throw his arm about suddenly and violently, the artery will be endangered. I should say then, that, in such circumstance, prefer the median cephalic vein; but if you cannot get blood from it, let the patient's arm be well fixed at all events, feel for the pulsation of the artery, and make the opening in a part of the median basilic vein not immediately over it. The great Dupuytren had seen so many accidents arise from bleeding in the median basilic vein, that, as his lectures will convince, he had a kind of timidity about bleeding in this vessel. With the precautions, which I have offered, however, it may be performed with sufficient safety.

2dly, This case should impress upon your memories the expediency and correctness of the great practical rule applicable to a recently wounded arterial trunk requiring to be tied; namely, always to expose, if possible, by an operation, the wounded portion of the artery, and to apply one ligature above, and another below the orifice in the vessel. Thus all risk of hæmorrhage from the freedom of the anastomosis is prevented.

3dly, If our reasoning and management of this case were right, you should prefer the ligature to compression, when the artery has been extensively opened, and the limb paralytic; because the first circumstance would make the healing of the arterial wound less likely to succeed; and the second would render the limb less able to bear compression, without being followed by gangrene.—*London Med. Gaz.*

*Poisoning with Antimony.*—Dr. Lohmeier, of Schönebeck, has related some interesting cases of poisoning with antimony which occurred to workmen employed in the manufacture of anatomical preparations. They were exposed, in their operations, to the vapours of oxide of antimony, antimonious and antimonic acids, and hydrochlorate of antimony. The symptoms were, slight pain of the head, with tightness of the chest, gradually increasing to pain and severe stitches, and accompanied with a dry, racking cough. To these symptoms succeeded swelling of the cervical glands; burning and lancinating pains at the back of the neck and head; scanty expectoration, accompanied with sibilous râles; nocturnal sweats, and diminished appetite. Diarrhœa, with griping pains, and enlargement of the abdomen, came on, and subsequently stranguary, pains of the testicles, with loss of desire, proceeding to actual impotence, and ultimately shrinking of the penis, and atrophy of the testicles. The symptoms, after disappearing under treatment, broke out afresh on the individual's being again exposed to the anatomical vapours. Dr. Lohmeier recommends local bleedings, and the administration of bark, for the treatment, and strong currents of air through the manufactories, for the prevention of the injurious effects to which the workmen are exposed.—*Casper's (Wochenschrift*, April and May, 1840;) *Edinburgh Monthly Journal of Medical Science.*

---

*Extraction of a Gold-Pin passed far into the Urethra.* By Dr. Boinet.—I was called some time since, to visit a young man, who, for the purpose of excitement, had introduced a gold-pin into the urethra, into which it suddenly slipped from his grasp and disappeared. It was more than two inches long, and the head which had been pushed in first was as large as a hemp-seed. In his attempt to push it out again he had made it go further towards the bladder, and when I came to him the head of the pin was in at the membranous part of the urethra. I could easily put it in the perineal region, and applying my thumb on the head to prevent its going on towards the bladder, I tried to push it out of the canal, pressing in the direction opposite to that by which it had entered, and at the same time pulling the penis, to prevent the point from catching in the folds of the mucous membrane. But, in spite of all my precautions, my attempts appeared to make it go towards the bladder, especially whenever I tried to disengage the point from the mucous membrane into which it kept running. I had scarcely any instruments with me, nor indeed would any ordinary ones have been of any use; therefore my endeavours to draw it out were unavailing.

I now determined to run the point of the pin through the wall of the urethra, and then to turn the pin end for end, and push the head towards the external orifice. This I accomplished in the following manner:—With the left thumb I firmly fixed the head of the pin, and then bending the penis double at the part where the point of the pin lay, I made the latter pass through the wall of the urethra, and drew out all but the head, which now lay where the head had just previously been. This done, I carried the shaft of the pin backwards, and so made the head move forwards, and then pushing on the shaft from behind forwards, I pushed the pin head first towards the external meatus, through which I now easily drew it out with a pair of dressing forceps. In a word, to perforate the urethra from within outwards, to turn the pin, to push it on, and to extract it—such were the manœeuvres of this operation.

The consequences of this perforation were of the simplest kind: the patient scarcely ever felt a pricking in making water. Three days after he was perfectly well. Subsequently, however, in consequence of a severe and maltreated gonorrhœa, an abscess formed around the urethra, and was followed by a fistulous opening; but it was remarkable that this opening was situated at a considerable distance from the part at which the urethra had been punctured.—*Lond. Med. Gaz., from Gazette Médicale*, Mai 1, 1841.

---

*Remarkable Cases of Hernia.* By M. DEMAUX.—At the meetings of the Anatomical Society of Paris, M. D. lately presented two remarkable preparations. The first was from a man advanced in life, of whose history nothing was known. M. D., in carefully dissecting a hernia which was situated at the right inguinal ring, and was as large as a moderately sized orange, met with some muscular fibres, which at first he knew not what to refer to, for the sac had not yet been opened. He soon found, however, that they were those of an intestine, and he then opened the abdomen, and found that the cæcum had slipped downwards, so that its posterior wall had come in contact with the orifice of the inguinal canal, through which it had formed a hernia. It was this posterior wall which presented externally. Within, a similar portion of the anterior wall formed a pouch, which was covered by peritoneum, and constituted a sac, into which a loop of small intestine had passed. On blowing into the lower extremity of the latter, the protruded portion of the anterior wall of the cæcum suddenly re-entered the abdomen, and with it the fold of small intestine which it contained. To have arrived at the latter in an operation, it would have been necessary to cut through, 1st, the skin and fasciæ, 2d, the posterior wall of the cæcum, 3d, the anterior wall of the peritoneum.

In the second case the patient was a man 56 years old, who had long had an inguinal hernia on the left side. For some time he wore a truss; but the rupture having ceased to come down he had left it off, and then, after long walking, the hernia re-appeared, and at last could not be reduced. The ordinary operation was performed, and it was scarcely necessary to divide the ring: a mass of omentum could not be returned on account of adhesions, but a small loop of intestine which lay behind it was replaced without difficulty. In three days the patient died of peritonitis. The orifice of the inguinal canal was found to present rather a large infundibulum; but it soon divided into two branches. The one which was anterior and internal extended down to the testicle, and still contained the mass of omentum; this was, therefore, a congenital hernia, and in this the strangulation had taken place: the other, posterior and external, though also very deep, contained nothing; its orifice was larger than that of the anterior sac, and would have admitted the finger.—*Ibid., from L'Examinateur Médical*, Juillet 11.

---

*Effects of Calculus in the Female Child.* By GEORGE A. REESE, M.R.C.S.—The following is the only case of the kind I have met with in the female out of nineteen thousand children who have been under my care; I consider, therefore, that if briefly recorded, it might be worthy of notice in your valuable Journal.

Ruth Mole, aged four years, was brought to me labouring under retention of urine, the mother stating that the child had not passed any water for two days and nights, and that the bowels had not acted during the same time.

July 12. There is considerable fever; great pain; constant moaning; the head hot, and tossed from side to side; the pulse small and frequent; the tongue dry, and covered with a brownish coating; there is some delirium; the abdomen is hot and tense; the bladder perceived to be much distended, extending up to the umbilicus; the external organs of generation are inflamed; the clitoris distended: the nymphæ slightly œdematous.

The distress of the child demanding immediate relief, a flexible catheter was introduced, and twelve ounces of turbid urine were drawn off, and an active aperient was ordered.

13. Immediate relief followed the abstraction of the urine, and the child slept for four hours. The bowels have acted twice freely; there is constant inclination to go to stool, and considerable straining causing the bowel to prolapse. No water has passed since yesterday; the bladder is again palpably distended, and the same state of the external organs perceptible, but the fever has much abated.

The prolapsus ani and the state of the external organs of generation so analogous to what occurs in boys with retention of urine from urethral calculus (in whom the erection of penis with œdema of its integuments are the principal symptoms) led to the suspicion that the cause of retention in this instance might be calculus, which suspicion was found to be correct, by the introduction of a probe into the urethra. It was therefore determined to leave the bladder as it was, unless urgent symptoms supervened, in the hope that the pressure of the urine might expel the stone from the passage.

14. The child is much the same in all respects, but the urine has dribbled away in small quantities since yesterday. The stone may be felt with a probe still lodging in the urethra. After a little trouble this was caught hold of by means of a small pair of common forceps, and brought forward to the orificium urethræ, through which its size prevented its coming without violence sufficient to produce laceration; a small incision was therefore made, as less likely to be followed by incontinence of urine, and the stone extracted.

15. All symptoms relieved, but there is incontinence of urine.

22. The child is free from all symptoms, the incontinence of urine having ceased for the last four days.

The calculus is five lines in diameter, weighs eleven grains, and is nearly perfectly round. I believe a calculus of any other shape *could* hardly produce such symptoms in the female child.—*Lancet.*

---

*Employment of Creosote for the cure of affections of the Eye.* By G. T. Black.—Two years since I suffered severely from inflammation of the eye, occasioned by a cold, and which, perhaps, from inattention, assumed a chronic form. Frequent attempts at removal were made; first by refrigerant and sedative collyria; then by astringents, zinc, nitrate of silver, &c., which produced but slight benefit. Accident, however, shortly did all that I desired—effected the cure.

Whilst replacing on the shelf a bottle of creosote, a small portion of that which usually lodges round the stopper dropped into the affected eye. For a few minutes the pain was extreme; the existing inflammation was also superadded to: these effects were only temporary, soon subsiding into ease, and a sensation of coldness in the eye was experienced. From this time the inflammatory action greatly weakened, till complete restoration took place; subsequently I reflected upon the palpable and sudden alteration for the better, and determined to put the means to the test of experiment, to confirm or annul the claim of the agent. The comparatively few cases, however, which have since fallen under my notice, or rather under my immediate care, do not justify my putting the remedy forward as a specific; yet, in the majority of ophthalmic cases, particularly when of a chronic nature, I believe none of the means now usually employed promise fairer, as a therapeutical agent, for their speedy removal. Three cases are enclosed, in addition to my own, in which I have been happy enough to succeed.

*Case* 1.—A man, aged 40, temperament nervo-bilious; symptoms much as usual, but severe; redness of the conjunctiva adnata, &c.; excessive pain, intolerance of light, and lachrymation; thirst; headach; preternatural heat of body; great pulsation of the temporal arteries, and other febrile symptoms:—venesection to twenty ounces; calomel jalap purges, abstinence, and poppy decoction. These but ill succeeded. Sedative lotions, tartar emetic in nauseating doses—slight benefit. Calomel and opium three times a day: vessels of the conjunctiva still distended, but less bright; general health better; less headach, &c. Patient thoughtlessly absented himself for two days: on next appearance the eyes apparently much the same, but complains of dimness of sight; stimulating lotion; no evident change. Creosote lotion* to be used three times a day. At the expiration of a week, less redness, and no dimness of sight. I have once seen him—quite recovered.

*Case* 2.—A young man, aged 18, (lymphatic temperament,) while at his usual employment had a piece of lime accidentally thrown into his eye, which produced inflammation, that ended in an ulcer near the internal canthus. Upon a subsequent attack from exposure to cold winds the cicatrix spread to a great extent, bordering on the margin of the pupil, attended with indications of chronic inflammation. As far as I conveniently could, the excrescence was removed by knife; the after-application of creosote, in an undiluted state, diminished the remaining portion, and dissipated the inflammation.

*Case* 3.—A child with *ophthalmia tarsi.* It yielded to none of the usually employed remedies, but gave way to the application of the creosote ointment, and the internal administration of the disulphate of quinine. I am fully aware how generally the therapeutical properties of creosote have been called into notice, such as an application to burns and ulcers, in herpetic, furfuraceous, squamous, and crustaceous skin affections; in bronchitis to promote expectoration; in toothach; in atonic rheumatism, &c. &c.; but I have not yet seen any account of its use in affections of the eye, which will, perhaps, excuse me, should such have been the case, for thus taking attention from more important matters.—*Ibid.*

---

*Dislocation of the Wrist.* By Ralph N. M'Dermott, Surgeon.—A young gentleman, ætat. between 14 and 15, climbing over a high

---

* The formula of the lotion employed, gradually increasing the strength according to circumstances, is

℞ Creosote, m. iij;
Compound tincture of lavender, m. xx;
Distilled water, ℥ss.

wall, and finding himself falling, instinctively put out his hands to break his fall. He came with all his weight on his out-spread palms, and states, that "his wrist was doubled under him," the inferior incisors cut deeply into the lower lip, and the left wrist was dislocated. I was sent for, and saw him in half an hour after the accident occurred. The carpus formed a tumour posteriorly, above which there was a depression.. Anteriorly, I could feel the ends of the radius and ulna, in the palm of the hand, which was semi-flexed, and supported carefully by his right hand. He complained of a numb or dead sensation in the limb.

Reduction was easily accomplished, and the power of motion in a great degree restored to the joint. A splint and cold lotion were applied, both of which were laid aside after the second day, not being found agreéable to my patient. A professional friend saw this case with me, and at once concurred in the diagnosis of dislocation.—*Ibid.*

We place the above on record, without attaching much value to it; we do not deny the possibility of dislocation of the wrist, but a fall upon the outspread palm so constantly produces a deformity similar to the above, dependent upon fracture of the lower extremity of the radius, a dislocation is so improbable under the circumstances, that we require more than the concurrence of a professional friend to induce us to admit it. The details upon which the differential diagnosis was founded, should have been communicated.

---

*Wound of the Brachial Artery in Venesection.*—M. W., late lieutenant R. N., admitted April 8, under the care of Mr. Quain, (dresser, Mr. G. Camrey.) It appears that about two hours ago, when going to the office with which he was connected, he was seized with a fit resembling apoplexy, and was taken to a surgeon, who states that he attempted to bleed him in the median cephalic vein, but failed, owing to its small size; and that while puncturing a vessel situated more internally, "the patient suddenly jerked his arm forward, and immediately a large gush of arterial blood flowed from the wound." A tourniquet was applied in the course of the brachial artery, and the patient brought to the hospital.

On admission the patient was stated to have lost about thirty ounces of blood, and when the tourniquet was slightly relaxed, a free gush of arterial blood took place, from a wound situated over the course of the brachial artery at the bend of the elbow. Mr. Quain determined on securing the bleeding vessel, by placing a ligature above and below the puncture. The patient being placed in a favourable position, and the arm supported on pillows, the tourniquet removed, and pressure made by the fingers of an assistant, while the venous blood was prevented from flowing by pressure on the forearm made with the hand of another assistant; the wound was enlarged to the extent of about two inches in the course of the vessel; and some coagulated blood being removed by means of a sponge and the handle of the scalpel, the tendinous expansion of the biceps muscle being brought into view, was divided to the same extent. The artery was now exposed, and an opening, occupying nearly half its circumference, was brought into view. Ligatures were then passed round, and secured one above, the other below the opening. The pressure being removed, all hæmorrhage had ceased. Lint, steeped in cold water, was then applied, and the patient placed in bed. He was almost insensible; pulse in the left forearm 60, and full; none in the right forearm; breathing slow, not stertorous; pupils much contracted; face drawn to the left side; right arm and leg partially paralysed. Ordered to have six grains of calomel immediately, and a drop of croton oil every two hours until it operates.

*Vespere.*—Continues much the same; wound closed by strips of isinglass-plaster; and the arm surrounded with flannel.

9. Continues in nearly the same state; bowels have been freely opened; he frequently raises his left hand to his forehead. Ordered ten leeches to the temples, and a blister to the nape of the neck. A feeble pulsation or thrilling can be felt in the radial artery of the right arm. He continued in nearly the same state until the 13th, when the report states him to be much worse. Pulse 130, small; constant rigors; feet cold. The discharge from the wound in the arm very offensive, and there is much ecchymosis around it. In the evening he became still weaker, and died.

*Post-mortem. Head.*—In the left ventricle of the brain was found a large quantity of dark coagulated blood; the substance of the brain around, including the thalamus opticus and and corpus striatum, was softened and disorganised.

*Chest.*—The lungs were much congested, and easily broken down; the mucous membrane of the bronchi presenting distinct marks of inflammation. The summits of both lungs were marked by old and firm cicatrices; and a few miliary tubercles were scattered through them.

*The Heart.*—The viscera of the abdomen were healthy.

*State of the Arteries of the Arm in which the Brachial had been tied.*—With the view of ascertaining with exactness by what branches the circulation had been restored to the forearm and hand, after the application of the ligature to the main vessel, common injection was forced into the end of the axillary artery, above the origin of the collateral branches which ramify about the elbow-joint.

On pursuing the examination, two ligatures were found on the brachial artery; one on each side of the wound, and the lower one at the distance of an inch and a half from the bifurcation of the vessel. The arteries above the situation of the ligatures, both the brachial and its branches, were much distended. Those below the ligatures, the end of the brachial, the radial, and the ulnar, though containing injection, were of less than the natural size, especially the last-named artery. This circumstance is doubtless owing to the difficulty of forcing a substance of considerable density through the small anastomosing branches.

The vessels by which the communication was established between the artery above the ligature to those below it were the following:—

In front of the joints the "anastomotica magna" joined the anterior ulnar recurrent by a long slender branch, and on the outer side the "superior profunda" and the radial recurrent were connected in the same way. The branch of communication in the latter case being on the ridge above the outer condyle of the humerus, and behind the attachment of the supinator longus. The branches in the situation of the musculo-spiral nerve were not seen to join.

On the posterior aspect of the limb the branches were of larger size, and they formed a network behind the joint. Behind the inner condyle of the humerus small branches of the "inferior profunda" running along the ulnar nerve, joined with equally small ones, derived from the posterior ulnar recurrent, the "anastomotica magna" crossed the lower end of the humerus, under the triceps muscle, and branching out largely, communicated freely with the superior profunda from above, and with the interosseous recurrent from below.—*Ibid.*

---

*Wound of the Palm—Secondary Hæmorrhage—Ligature of the Brachial.*—J. C., aged thirty-six, a strong, healthy, labouring man, was admitted June 15, under the care of Mr. Liston, on account of injury to the right hand, produced on the 13th instant, by the explosion of a powder-flask, which appeared to have forcibly torn asunder the palm of the hand. The hand was much scorched and blackened by the injury, the fleshy part of the ball of the thumb being deeply lacerated, and the thumb itself almost thrown upon the forearm. The pain was severe, affecting the whole hand and forearm. The bleeding was comparatively slight, and soon ceased altogether. The parts had been replaced by a surgeon, who attended until the patient was admitted into the hospital.

On his admission he was suffering from violent inflammation, which had already commenced in the injured parts. The whole hand, but more particularly over the outer and back part of the thumb, was much swollen, hot, and excessively painful. The inflammation extended along the wrist and lower part of the forearm. A deep laceration existed between the thumb and forefinger, which appeared to extend nearly down to the carpal end of the first metacarpal bone. The wound was blackened and sloughy, with a thin, bloody, and offensive discharge. A litle behind this laceration another longer but more superficial one appeared, which extended from nearly the middle of the palm, round the root of the forefinger, to the dorsum of the hand. Patient's tongue dry and coated; bowels confined.

He was ordered to keep in bed, with the hand and forearm well elevated; water-dressing applied to the wound; the forearm frequently fomented. To have a dose of colocynth and calomel at bedtime, and an aperient draught to-morrow morning.

16. The hand much less painful; swelling and tension greatly reduced; less thirst and fever; bowels well opened.

17. Patient appears much easier.

19. Still doing well; wound discharges abundantly; the sloughs are separating, and granulation has commenced; the thumb will now admit of closer approximation by bandage.

21. Wound much cleaner, though there are still some sloughs at the deeper parts to separate. A lotion of diluded chloride of soda to be applied to the wounds.

23. This evening the house-surgeon, Mr. Potter, was called down on account of hæmorrhage, which had been profuse, probably to the extent of sixteen or eighteen ounces; the patient was pale and faint. The bleeding, which had ceased on his arrival, was apparently arterial, and was described by the patient as coming out in jerks. A graduated compress was placed in the wound, and secured by bandages, applied over the fingers and hand, to as high as the middle of the forearm. The parts to be elevated, and cold water applied frequently to them.

25. No return of hæmorrhage; hand not at all painful.

27. The bandages were found slightly moistened with blood; hand rather painful, and throbbing.

In the evening the house-surgeon was again summoned to him, on account of great pain, which had come on suddenly, and was rather increasing. He described it as of a throbbing, bursting character, and causing the bandages to appear tight. On removing these and the compress no bleeding occurred, but an elastic, rounded, pulsating swelling was detected on the outer aspect of the hand, between the first and second metacarpal bones. There was no redness of the skin over the tumour, although the back of the hand appeared somewhat inflammed. The swelling was elastic and compressible, but on removing the pressure instantly regained its rounded form. Pulsation felt over a space of about one and a half or two inches square, and as evident on lateral pressure. On compressing the radial artery the swelling diminished considerably, and instant relief followed. It was evident that a diffused, false aneurism, of the termination of the radial artery, had occurred.

Mr. Liston having been sent for, arrived at about half-past eight in the evening, and immediately proceeded to put a ligature round the brachial artery, by making an incision over the inner edge of the biceps muscle, about three inches in length. This divided the skin and fascia: the biceps was then drawn to the outer side, and held by a retractor. The medium nerve was thus exposed, and the cellular membrane over it having been divided, it was drawn to the inner side by a blunt hook. The vessel was then completely displayed, together with its venæ comites. The artery was now detached from the veins, and from its sheath by a few touches of the point of a scalpel, and a strong silk ligature passed round by an aneurism-needle. When this was tightened the pulsation in the tumour ceased altogether, and the pain was aleviated. The edges of the wound were brought together, and retained by two points of sature and isinglass plaster.

Mr. Liston observed, that this operation was performed on similar principles to those by which secondary hæmorrhage from stumps was arrested, viz: by tying the main artery of the limb, and thus lessening the flow of blood to the part. Experience had shown that it was not necessary in these cases to apply a ligature close above the bleeding point; and it was fortunate that such was the case, because in most instances it would be impossible to decide beforehand what vessel was bleeding, and the attempt to search for it in the middle of indurated tissues, or in cellular membrane gorged with coagulated blood, would be absurd. In the present case no one would attempt to find the bleeding point in the palm, because the tissues would be so much altered by inflammatory action and effused blood, and there would be a mere fistulous track leading to the vessel, which could not be drawn out nor secured.—Ligature of the radial artery might succeed in some cases of secondary hæmorrhage from the palm, but it was always a very uncertain remedy, owing to the very free anastomosis of the ulnar artery; and in the present instance the pulsation in the tumour was only partly arrested by pressure on the radial. Ligature of the radial and ulnar arteries was unnecessarily severe, as it made it necessary to make two incisions instead of one, and that, too, without the insurance of perfect success; inasmuch as the interosseous artery would still supply the same quantity of blood. By applying a ligature round the humeral artery, the circulation through all the vessels of the forearm was lessened, and this experience had shown to be quite sufficient assistance to nature in remedying accidents like the present one.

Mr. Liston also remarked, that he looked on this as a point of considerable practical importance in wounds of the superficial palmer arch, &c. He believed in these cases that when the bleeding could not be commanded by methodically applied pressure, it was much better to tie the brachial artery at once, than to apply a ligature to only one of the vessels of the forearm, with the chance of being obliged to tie the other in a day or two; and even then, not being safe from future hæmorrhage.

29. No bleeding from the wound in the hand, which has resumed a healthy appearance: the tumour has entirely disappeared. The wound caused by the operation has united in the greater part of its extent. The sutures were removed, and warm-water dressing applied. The circulation in the arteries of the forearm is already restored, the pulsation in the radial being perfectly natural.

July 5. The wound of the hand has made great progress; suppuration very copious. The incision in the upper arm was united by granulation, leaving a small fistulous opening for the ligature.

19. The ligature was pulled away this morning, having been firmly retained until now, probably, by the knot entangled in the granulations. The circle of thread which had embraced the vessel was so small as hardly to admit a pin's-point.

August 1. The wound in the hand being now firmly united, the patient was discharged. —*Lancet.*

---

*Formidable aneurismal state of the leg cured by compression.* By SAMUEL YOUNG, Esq., Surgeon.—Dec. 18, 1839. Rebecca Thomas applied for a curious vascular affection of the left leg; from an aneurismal spot a pendulous body issues at the outer part of the limb, three inches below the knee. The patient is full habited, and of a florid, sanguineous temperament; age, thirty years. First noticed the disease ten years since, in consequence of receiving a slight blow on the part; from the immediate pain produced was then first led to examine the part, and there found a dark, purplish crimson spot, about the size of the top of a woman's thimble. The spot at first was flat on the surface, and its covering like a very thin vellum, but soon rose up to the height of half an inch, hot and pulpy to the feel. On this occasion, after the pain, which continued for some ten minutes or more, went off, the tumour which had so risen returned to the level of the skin. For six years it remained without any apparent growth or pain, except at the latter part of this time, when it remained much longer above the surface than in the first mentioned instance, after accidentally receiving the slightest touch.

On the Thursday before Christmas-day (1836,) whilst stooping to draw off a person's boot, she felt something very warm trickling down the leg, and on looking, perceived blood on the ground and her clothes drenched. The part was bandaged up with flour, and the patient went to bed, when the bleeding ceased; next day she applied to a medical man, the part was swollen full two inches above the surface, and was more than an inch and a quarter in diameter at the top.

In the course of three or four days the tumour subsided; but from the bottom the present pendulous substance was produced, which is now fully the size of a large fig, and largely supplied with blood-vessels. At the time, however, of the subsiding of the tumour, this appendix was only the size of the first joint of a moderate forefinger; the very dark colour of the tumour then subsided.

The fig-like appendage grew rapidly the first twelve months to nearly its present size; even in a fortnight after the subsiding of the tumour as here described, upon the appendage being roughly handled at a surgical examination, a physician aud surgeon being present, the blood was thrown upwards with great force on and over the patient's shoulder, and continued bleeding largely from the lower edge till bound up with plaster straps and lint compress.

Next day, when the applications were removed by the medical attendants, on being again handled, the fig-like appendage not only swelled up, but the aneurismal skin also rose full three inches above the surface. The patient describes the feeling at the time as if the whole were forcibly being dragged out by a pair of pincers.

At the same moment, from the under and lower edge of the appendage (which the patient herself calls the *tongue*,) an immense hæmorrhage commenced, filling a large wash-hand basin half full. The bleeding was of that character and to that amount, as to induce the physician who was present to exclaim, "By ——— the woman will bleed to death." The patient was rendered so weak in consequence, that an hour after it was bound up she could not walk without assistance.

About this time it was that one of the many surgeons who attended upon the case proposed removing the parts by the knife, describing a large area on the leg around and considerably beyond the disease with his finger; this was objected to, and evidently most wisely, by the two professional gentlemen just alluded to; they preferred the removal of the limb, rather than hazard so uncertain an operation, and urged its adoption at once—the fear being at the time that the patient might sink if a second bleeding took place during the night. Other long-established practitioners also, in after consultations, agreed in the necessity of the removal of the limb for the ultimate object of saving the patient's life.

For a month after this profuse bleeding, upon every removal of the applications, it bled slightly from the under edge of the appendix; but in a few days after the great hæmorrhage, the aneurismal spot itself again subsided to the level of the skin.

Some twenty months since, after kneeling, the knee and leg swelled, and turned black in spots as if pinched. The following morning some active bleeding came on, wetting through the linen and roller, but nothing to be compared to the former, since which no bleeding of any consequence has occurred; but the leg has gradually increased to its present state, spotted all over with purplish cutaneous veins, with immense enlargement, and particularly about the outer as well as the inner ankle, and spreading to the foot, which it so involved as to leave but little trace of natural shape.

In way of remark, it may be observed that the disease in question is in its nature somewhat anomalous; though decidedly of that description which John Bell was the first so accurately to describe under the head of "aneurism by anastomosis," in some respects it has the feature of fungus hæmatodes, but nothing whatever, in the most distant degree, of the *mere* varicose vein.

In so enlarged and distorted a state of the limb, relative situation of parts is in a great measure disturbed, if not wholly lost; but still the situation of the aneurismal spot, one would say, is just within the inner edge of the peranæum muscle, and the outer edge of the tibia; that is, just over and about the tibial artery and vein: a most suspicious circumstance, for, from the *erectile* nature of the aneurismal substance, as proved by the history of the case, there can be no doubt but that a highly vascular and spongy tissue of considerable extent must form its basement. In such a neighbourhood, to what extent, and how formidably supplied by blood-vessels, is the question. Great uncertainty and risk would, therefore, necessarily attend the dissecting out, or, rather, *the attempt* to dissect out, such a disease; and the surgeon, as well as the patient and others, must be all prepared for the possible, if not probable, immediate amputation of the limb to save life in case of failure.

*Treatment.*—Dec. 19. The fig-like appendage was left as a guage to show how far the vascularity of the aneurismal spot and its basement was affected under pressure; otherwise, had it been deemed prudent, a ligature might have been applied to its pendulous neck.

The general restoration of the limb to health was, however, the first object; for this purpose the foot, heel, ankle, and leg, were accurately rolled, at least as well as the great irregularities and enlargements of the parts admitted; active purgatives and calomel in alterative doses were prescribed.

25. Rolling has been followed up; at times interrupted by occasional swellings of the foot, &c., at night. However, gradual increase of pressure has still been persevered in, and the limb to-day presents a remarkably improved state, both as to size as well as the diseased and discoloured appearance of the skin and its veins. The pain she used to suffer has almost subsided, especially from the aneurismal spot down to the inner ankle, where the enlargement was deeply discoloured; all this is now nearly removed, and the enormous bulk outwardly above and over the ankle and foot is also strikingly reduced. Very active pressure employed; the purgative plan continued; health improved.

After the second application of compression the aneurismal spot and appendix were placed under the pressure of plaster straps; to these, additions have been made, and over them a firm, graduated linen compress has been included in the application of the rollers.

Jan. 3, 1840. Very active pressure has been kept up from time to time since last report; no pain or swelling have occurred. To-day, upon the removal of all the rollers, the limb appeared considerably smaller than the other leg; above the ankle, where it was so enormously enlarged, a full inch less by measurement than the other leg, the integument loose and flaccid.

Specific pressure employed over the aneurismal spot and fig-like appendix by graduated compress paper and plaster straps; the former strap applications not being removed, and the whole limb placed under very active pressure, but more especially over the immediate disease by the further employment of linen compress and the firmest rolling, the pressure being gradually carried up to the uttermost drawing the hand and arm could use, assisted also by the knee. In this process of the treatment, pins were used as fixed points to confine the specific pressure to the part, and prevent ligature on the limb.

12. None of the plaster straps have been removed, so that the immediate state, either of the aneurismal spot or appendix, has not been ascertained. No pain or inconvenience of the part has been experienced; but evidently a diminished circulation to a great extent has been effected, from the different sensation felt by the patient herself, as well as the total disappearance of large vessels beneath the integument, which formerly were seen passing along the upper and outer part of the leg to the thigh, some inches above the aneurismal spot.

19. To-day, on attempting to remove the plaster straps, the fig-like process was unfortunately dragged, which caused considerable irritation to the aneurismal spot, and which, from a perfectly quiescent and reduced state, rose frightfully up on the instant, giving a dreadful sensation of tearing and bursting of the whole limb.

This circumstance, which gives a tolerable reproof to those who would, in their simplicity, treat such a case as *a nothing*, precluded anything like further inspection, and the parts were immediately placed under the control of the compression: this was cautiously and gradually increased over the aneurism to a maximum height, and in the course of less than a minute all the dreadful sensation and pain subsided.

Feb. 23. So much amendment in the case, that re-applications are now only made once a-week. No inconvenience experienced in the limb; the cuticular veins have altogether disappeared: and the patient observed, "that it was a long time since she enjoyed such health as she now feels." Active pressure kept up.

March 1. Walked into town five miles to have the applications made; and more than a fortnight since the patient walked ten without the slightest inconvenience. Before the treatment, could not walk half a mile without being ready to drop from pain. Upon removal of the pressure immediately over the aneurism, the parts have now none of that filling, bursting sensation, which formerly always attended upon every such removal; this sensation has entirely subsided the last three weeks. Active pressure continued.

April 12. To-day all the straps were removed, and particularly the two between which the neck, as well as part of the process, were included; part of the surface had been slightly abraded, and from it a small discharge had issued, staining the rollers immediately in contact. On removal of the two straps from above and below the neck of the process, the two blood-vessels passing through it into the body of the process soon began to fill and swell up the part, which before appeared quite flattened, thin, and apparently bloodless; the small abraded surface became covered with minute scarlet points, and blood, quite of an arterial character, soon began to drop freely and fast from it, but not attended with any jet. During all this, the aneurismal spot itself remained flat—indeed, concave in its surface.—Before the re-applicication of the compression which was made immediately over the part, some two ounces of blood fell upon the carpet; after the application of the compress there was no stain of blood after the first coil or two of the roller.

May 10. The fig-like process, from the discharge and fœtor, would appear to be in a sloughing state; but all the applications immediately in contact were not removed from their adhesive state, and also, in consequence of the removal of a part, some oozing of blood took place; the remainder, therefore, were left, as there was no object for their removal, but, on the contrary, such would only encourage a return of circulation through the part by the absence of pressure.

23. The discharge on Saturday last was extremely fœtid, and the compresses in consequence over the aneurismal spot were all removed, and in a part, also, over the fig-like process. To-day, the compresses had slipped down, leaving the aneurismal spot free from all pressure; but no filling of the part as formerly was at all perceptible; and, on the removal of all the compresses, the once fig-like process (or tongue) which had caused so much anxiety to the patient for years, on account of its frightful swellings and bleedings, was now found among the black, fœtid discharges quite disengaged; a mere dark, putrid mass, about the thickness of half-a-crown; a small pedicle of the elongated neck only remaining at the bottom of the aneurismal spot. The limb was placed under general compression.

30. The applications had slipped down today, so as to form a ligature across the aneurismal spot; but no swelling or rising of the under part took place, though the covering was of a dark purplish colour, evidently in consequence of the ligature. On the reapplications being made, more specific compresses and pressure were placed immediately over the aneurismal spot and pedicle. The limb had remained perfectly easy and comfortable during the week, though the last few days the patient had suffered from an old spasmodic stomach complaint, for which remedies were prescribed.

June 4. All the applications removed, and still more active pressure used in the re-applications. The stomach attack less the last few days.

12. The whole of the applications removed, and the remaining pedicle or neck appeared in a sloughing state, giving out the same putrid discharge as the fig-like process did when in a state of slough. Active compression over the aneurismal spot and pedicle continued; and which is effected, including the whole of the leg and foot, by three five-yard rollers; formerly thirty-three yards required to be used. The patient's general health much improved.

July 19. The covering integument of the aneurismal spot and appendage this day presented a perfectly smooth and delicate cuticle, marking the progressive change going on from the last report.

Aug. 9. The cuticle over the spot and appendix rough and scaly. In speaking of the former state of the diseased leg, as compared with its now wonderfully changed condition, the patient described the effect of cold, at times, as well as heat upon the disease; and observed, "that people, even medical gentlemen themselves, who only saw it in its quiet state, could have no idea of its frightful swelling and alarming appearance at times;" and named an instance, "the winter before last, after being long exposed to cold, of the sudden and enormous swelling of the leg itself (holding both her hands out with extended fingers in a cradle-like form, to show the size of the limb, and which would have taken in the body of a well grown child of two years,) as well as the rising of the aneurismal spot in a frightful mass of several inches—so frightful, that her mother as well as her friends were distracted and alarmed, and ran out of the room, thinking it would burst."

Jan. 10, 1841. Since the last minute the parts gradually subsided into a state of health, and the compression diminished to mere rolling, sometimes the patient applying the rollers herself. About six weeks back, the limb was tested by a desperate fall in consequence of her patten catching an iron railing, which threw her violently on her side and bruised her dreadfully. The leg was swollen and discoloured outside, and the veins enlarged; but no appearance of enlargement or pain was felt about the part, which was once the seat of so formidable a disease.

Feb. 21. Since the last report the leg had been seen but once, the patient managing the rollers herself, which are now employed chiefly for the recovery of the integument of the outer ankle, which formerly was so enormously distended, but which has now nearly recoved its natural state; the leg otherwise being in a perfectly healthy condition, the patient observing, "I really think it stronger than the other." To-day's report also affords a gratifying confirmation, that throughout all the late extremely cold and boisterous weather the limb has remained in every respect perfectly easy and unaffected, though the patient has been much exposed and out in all weather. So far, then, as testing the case in almost every possible way, by extremes of heat and cold, and severe straining and bruising as well as strong exercise, it may be said to be entirely a successful one. The patient is in excellent health.

The entire command of the compression treatment over the "aneurism by anastomosis," which has so often proved fatal in children, commonly known by the title "mother's marks," has been also proved, since the one so cured in 1828 and 1829, in the instance of Mr. Kennerly's child, the engraver and artist of London. This was a frightfully formidable case of considerable bulk and height, and situated upon the windpipe. One of the pressure plates used in this case the author has now by him; it measures full two inches across, and nearly as much in diameter; made up with tea lead and plaster straps to the thickness of three quarters of an inch, and is of the firmest texture, the internal plaster bearing the marginal mark of the aneurism.

Before it came under the treatment of compression, a consultation was held upon the case by Messrs. Lawrence, Vincent, and Stanley, surgeons of St. Bartholomew's Hospital, who declared the fatal nature of the disease, and the little, if any, chance of saving life even in the attempt at removing it by the knife. Some four years after (1833,) the author was met by the uncle of the little patient, who told him she was grawn a fine bouncing girl, and with scarcely a visible mark left of the former disease.

*Ibid.*

*On the exhibition of small doses of mercury in effecting Ptyalism.* By Charles Clay, Esq., Surgeon, Piccadilly, Manchester, Lecturer on Medical jurisprudence, &c. &c.—In the Lancet of January 18, 1840, is reported a discussion on the effects of small doses of mercurial preparations in producing ptyalism, and some valuable hints are thrown out on that subject by Mr. Snow. I think it will be generally admitted by all medical men of lengthened experience, that if ever the homœpathic principle was justifiable, it would be found in the exhibition of mercurial preparations. The great variety of opinion expressed as to the plan and dose to be given to effect the same object, stamps the whole with doubts and surmises which it would be well to remove, if possible, and place the subject on a more certain basis. There is not anything more certain than that ptyalism has been produced in very many instances by an almost incredibly small quantity of mercury in some form, whilst it is equally certain that amazing quantities of a similar preparation have been given without the slightest effect being produced. Many attempts have been made to explain this discrepancy, but as yet unsatisfactorily. Mr. Snow attributes the success of small doses in producing ptyalism, not to an idiosyncracy of constitution, but to the existence of acidity in the first passages; and he was led to form this opinion from the rapidity with which mercury affected individuals who happened to be taking at the time acidulated mixtures, which appears extremely probable. It is also well known that habitually constipated habits are sooner affected with mercurials than those of a contrary tendency. Whilst I am writing these remarks, a lady under my care had only taken six grains of the blue pill before ptyalism was complete; and I have seen many cases where the same effects have been produced by even less doses. Dr. Law frequently produced ptyalism by one or two grains of calomel, divided into twelve or twenty-four doses, that is one-twelfth of a grain of calomel every hour; the full effects were often produced within the twenty-four hours.

In illustration of the same fact, I was some time ago consulted by a gentleman in consequence of three large, foul-conditioned chancres, showing no disposition to heal, although he had been under medical treatment some time, and had taken five grains of blue pill night and morning, for three weeks, with mercurial dressings and lotions of nitrate silver; he was much debilitated by a constant purging. I ordered him to discontinue the lotion, but to go on with the dressings, and in lieu of five grains of blue pill night and morning, I ordered five grains of blue pill to be divided into thirty pills, mixed with some simple material, and gave one three times a day. The purging soon ceased; on the third day the mouth was affected, and on the tenth day the sores were healed. There cannot be a doubt but that the acid added by druggists to the conserve of roses, adds to its efficacy more than the knavery of the act contemplates at the time; and it is also very probable that the efficacy of the blue pill is still further increased by the druggists not putting into the mass the full amount of mercury, thus causing practitioners to exhibit it in much less doses than it is intended, and by this confirming the proposition of the small dose theory. On the supposition of acidity in the first passages, Mr. Snow hints at the probable usefulness of the bichloride, as a means for producing ptyalism, having an acid combined with it. Now, although it may appear a startling assertion, yet, with all deference to Mr. Snow, I scarcely *if ever* saw a decided case of ptyalism from the exhibition of the bichloride alone; and whenever I wished to produce that effect, I had invariably to give it in some other form, as calomel, blue pill, &c. I do not advance this on a few cases; I have observed it for twenty years past, and have pushed the exhibition of the bichloride to a very considerable extent, both in adults and children: in the latter of which acidity of the first passages prevails almost universally, independently of that contained in the preparation itself. It is on the result of extensive trials that I presume to differ in opinion with Mr. Snow, that the bichloride would be an improvement in producing the effects ptyalism rapidly; for I believe that of all mercurial preparations the bichloride has the least tendency to produce the effect, even when the system to which it is applied appears in every way favourable for the exhibition. It may not here be out of place to inquire how far the common custom of taking large doses of the blue pill in dyspepsia, and other fashionable (and of course) prevailing complaints of the digestive organs, is justifiable.

When it is recollected that one of the greatest ornaments of the medical world was instrumental in introducing that practice, now so commonly adopted without the least reflection as to the consequence, if it should happen to produce a different result to the one expected (viz. the taking of five or ten grains of blue pill at night, and a black draught next morning,) it is necessary to be a little cautious in combatting such high opinions; yet it has often struck me as remarkably absurd, *first*, to conclude that a mercurial preparation is indicated for its specific action on the digestive organs in restoring them from a diseased to a healthy condition; and, *secondly*, to give it in a dose so large as to act only as a simple purgative, which action is rendered still more certain by giving an active purgative afterwards.

Now I maintain all that is here effected might have been accomplished by simple and judicious purgatives. On inquiring why the active purgative is given, the answer usually is, to prevent the unpleasant consequences of accumulation of mercury in the system, producing worse consequences than the disease intended to be corrected. If this be really the case, why not give it in small doses, and narrowly watch its effects, so as in sufficient time to check any such tendency. The consequences of the *abuse* of mercury are often so dreadful, that its *use* should be based on the best possible principles. If mercury has any peculiar and specific action, it necessarily follows that it is either *called for or not* in that the treatment of disease. *If it be called for*, why is it given in such doses that its specific action does not manifest itself, producing an effect only that can as easily, and with much less risk, be accomplished by more simple agents. *If it be not called for*, why is it to be given at all, when simpler means would accomplish all that is required. There is scarcely anything more common than for individuals in respectable society (who, of course, ought to be well informed,) to swallow five or ten grains of blue pill, and frequently repeat the dose, without reflecting on the consequences, even on the slightest possible derangement of the stomach; whereas a little curtailment of their usual luxuries, or at most a simple purgative, would be quite sufficient. This all potent remedy too often fails in that class of diseases in which some consider it specific. I am myself an advocate for its use, but can see that it is much oftener abused than used with effect. I was consulted some time ago by an over-wise gentleman, who had practised upon himself freely by taking blue pill on every trifling symptom brought on by his own indulgences, until it had no effect, except as a purgative, as he had always taken it in six grain doses. I substituted half grain doses, and all the good effects of the mercury, as a corrective, were immediately apparent. The worst symptom, however, still remained; viz., too frequently indulging in his usual luxuries. From these remarks, I am led to believe the general exhibition of mercury is in far too large doses for securing its specific effects on those diseases to which it is applicable, and on this principle I give it in very small doses, and the form I generally administer is,

℞ Blue pill, grs. v;
Castille soap,
Liquorice powder, aa, ℈j. M.

Divide into twelve pills.

Sometimes I substitute calomel for blue pill: this appears sufficient for procuring the full specific powers of mercury. In respect to the producing ptyalism, valuable as the bichloride is as a medicine, I conceive its powers in that respect overrated; and it appears to me questionable if it ever produces it in the least degree. It would be desirable to ascertain if some other acids, in combination with mercury, would not be more favourable to such an effect than the muriatic. The facts, of first observing its effects more rapidly whilst taking acidulated mixtures, (which are generally sulphuric;) secondly, that acetic acid is as prominent as any other in the first passages; thirdly, the well-known effects of the compound calomel pill, where the sulphuric is equally prominent with the muriatic, would lead me to conclude that any combinations of mercury with acetec or sulphuric acids, would be more likely to produce the wished-for result than muriatic.—*Lancet.*

---

*Division of the Genio-hyo-glossi Muscles for Stammering.* By Alex. J. Lizars, M. D., Lecturer on Anatomy and Operative Surgery.—P. M., aged 35, had stammered from his infancy. The difficulty was evidently caused by spasmodic contraction of the muscles of the tongue and neck. The tongue, upon examination, was found to be shorter than natural. I operated in presence of my assistants, Messrs. Riccard and Hole, and two of my pupils, Messrs Collyns and Tibbets.

The instruments employed were, a straight sharp-pointed bistoury; a curved probe-pointed bistoury, with the cutting edge about an inch long, the remainder of the blade being blunt; a four-headed sling, or roller, and a compress of lint.

The patient having been placed in the sitting posture, with the sharp-pointed bistoury I made a puncture, rather less than a quarter of an inch in length, through the integuments of the lower part of the chin, about an inch posterior to the symphysis. I then pushed the curved bistoury gently upwards and a little forwards, until I saw its probe elevating the mucous membrane of the floor of the mouth; placing the forefinger of my left hand upon the probe-point and mucous membrane, I turned the cutting edge of the instrument to the right, and divided the muscles of that side; the bistoury was then carefully brought back to the mesial line, and the other muscle having been divided in a similar manner, the instrument was withdrawn. The compress of lint was then placed on the wound, and the four-headed sling applied in the same way as is done for fracture of the lower jaw.

Very little blood was lost during the operation; and after its completion the hæmorrhage was entirely stopped by the compress and bandage. Every thing went on favourably; the bandage was removed on the third day, by which time the wound had healed; and the patient resumed his usual occupation on the fourth day.

Immediately after the operation the patient experienced no difficulty in speaking, and the same has contined since. Upon examining the mouth after removing the bandage, blood was observed beneath the mucous membrane in the line of the submaxillary ducts; this was absorbed by the tenth day, and the patient was completely cured.—*Ibid.*

